# Blood Lead Levels and Cause-Specific Mortality of Inorganic Lead-Exposed Workers in South Korea

**DOI:** 10.1371/journal.pone.0140360

**Published:** 2015-10-15

**Authors:** Min-Gi Kim, Jae-Hong Ryoo, Se-Jin Chang, Chun-Bae Kim, Jong-Ku Park, Sang-Baek Koh, Yeon-Soon Ahn

**Affiliations:** 1 Graduate School of Medicine, Wonju College of Medicine, Yonsei University, Wonju, Korea; 2 Departments of Occupational Medicine, Dongguk University Gyeongju Hospital, Gyeongju, Korea; 3 Departments of Preventive Medicine, School of Medicine, Kyung Hee University, Seoul, Korea; 4 Department of Occupational Medicine, Dongguk University Ilsan Hospital, Goyang-si, Korea; New York University School of Medicine, UNITED STATES

## Abstract

The objective of this study was to identify the association of blood lead level (BLL) with mortality in inorganic lead-exposed workers of South Korea. A cohort was compiled comprising 81,067 inorganic lead exposed workers working between January 1, 2000, and December 31, 2004. This cohort was merged with the Korean National Statistical Office to follow-up for mortality between 2000 and 2008. After adjusting for age and other carcinogenic metal exposure, all-cause mortality (Relative risk [RR] 1.36, 95% confidence interval [CI] 1.03–1.79), digestive disease (RR 3.23, 95% CI 1.33–7.86), and intentional self-harm (RR 2.92, 95% CI 1.07–7.81) were statistically significantly higher in males with BLL >20 μg/dl than of those with BLL ≤10μg/dl. The RR of males with BLL of 10–20 μg/dl was statistically higher than of those with BLL ≤10μg/dl in infection (RR 3.73. 95% CI, 1.06–13.06). The RRs of females with 10–20 μg/dl BLL was statistically significantly greater than those with BLL <10μg/dl in all-cause mortality (RR 1.93, 95% CI 1.16–3.20) and colon and rectal cancer (RR 13.42, 95% CI 1.21–149.4). The RRs of females with BLL 10–20 μg/dl (RR 10.45, 95% CI 1.74–62.93) and BLL ≥20 μg/dl (RR 12.68, 95% CI 1.69–147.86) was statistically significantly increased in bronchus and lung cancer. The increased suicide of males with ≥20 μg/dl BLLs, which might be caused by major depression, might be associated with higher lead exposure. Also, increased bronchus and lung cancer mortality in female workers with higher BLL might be related to lead exposure considering low smoking rate in females. The kinds of BLL-associated mortality differed by gender.

## Introduction

Millions of people worldwide are exposed to lead. Lead concentrations in the general population have declined over the past two decades [1]. Blood lead levels (BLLs) were investigated in 13,043 Korean, lead-exposed workers from 1,217 companies in 2003 [2]. The geometric mean of the BLLs observed in these investigated workers was 6.08 μg/dl, or 1.6 times greater than the BLLs found in the general population (3.73 μg/dl) in 2002.^3^ 56.6% of these lead-exposed workers had BLLs over 5 μg/dl [2], 7.8% had BLLs over 25 μg/dl, and 0.9% had BLLs over 40 μg/dl [2]. Of the female workers, 33.8% who were investigated had BLLs greater than 5 μg/dl. The male workers showed relatively higher BLLs than the women [2].

The issue of lead carcinogenicity is of great current interest in science and public policy [3]. Although lead has been shown to be carcinogenic in laboratory animals, epidemiologic studies of occupational exposures have been inconclusive and the relationship between lead exposure and human cancer has remained unclear [4]. In 2004, the IARC Working Group reclassified inorganic lead compounds from ‘possible’ to ‘probable’ as human carcinogens (group 2A) based on limited epidemiological evidence related to an increased risk of lung, stomach, kidney, and brain cancers. [1].

Moller and Kristensen conducted a population-based survey of 1,052 men and women born in 1936 and living in Cophenhagen [5]. They found a statistically significant association between total mortality and BLL [5]. Gwini et al. found evidence of associations between occupational lead exposure and total morality and incident cancers of the esophagus, liver and bladder in Australian workers [6]. In the general population, individuals with BLLs of 20 to 29 μg/dl experienced cardiovascular, cancer and all-cause mortality compared to those with BLLs less than 10 μg/dl [7]. Menke et al. reported a significant relationship between BLLs below 10 μg/dl and cardiovascular and all-cause mortality [8]. Some studies showed that lead exposure was associated with cause-specific mortalities even with very low BLL [8,9], but little or no association was observed in other studies [10,11]. So, clear associations between BLLs and cause-specific mortalities have not been fully elucidated until now.

Since 1972, lead-exposed workers in Korea have been required to receive an Annual Specialized Medical Surveillance (ASMS) under the Industrial Health Safety Act [12]. This medical surveillance data was available for use in the construction of a cohort of lead-exposed workers since 2000. To our knowledge, no study has examined the association between disease-associated mortality and morbidity in lead-exposed workers in South Korea. The authors have explored the relationship between lead exposure and specific causes of death (exploratory analysis) to determine future research directions. In this paper, we explore the association between BLLs and cause-specific mortalities in male and female lead-exposed workers.

## Materials and Methods

### Ethics Statement

This study was conducted with the approval of the Institutional Review Board (IRB) of Dongguk University of Gyeongju Hospital (11–23). No information used to write this paper was from a hospital medical record. The cohort was constructed in 2011 and followed the mortality through the death registry of the Korea National Statistical Office (KNSO). So, the IRB of Dongguk University waived written consent of individual workers or their next of kin. In Korea, the follow-up of a previously constructed cohort before Nov, 2012 allows waived written consent, based on the premise that researchers protect the personal information of all study subjects. The protocol of this study was neither illegal by Article 18(②(4)) of the Personal Information Protection Act (PIPA) in Republic of Korea nor was there an ethical problem in collecting data for cause of death. In the Republic of Korea, the PIPA was activated April 1, 2012; the collection of personal data was not illegal before April 1, 2012. Also, by Article 18 of PIPA, a public institute can give a 3rd party information from statistical surveys and academic studies without a personal identification number. (http://www.law.go.kr/lsSc.do?menuId=0&subMenu=1&query=%EA%B0%9C%EC%9D%B8%EC%A0%95%EB%B3%B4%20%EB%B3%B4%ED%98%B8%EB%B2%95#liBgcolor0) (Korean).

### Study population

The ASMS data for exposure to occupational hazards (143 chemicals, 6 dusts, 8 physical agents and 19 metals including lead) including lead has been electronically stored and monitored since 2000 by the Korea Occupational Safety and Health Agency (KOSHA). The ASMS have been conducted for all Korean workers exposed to lead in the form of dust, fumes, etc., by more than 100 nation-wide medical centers approved by KOSHA through its quality control program for lead analysis in blood. The ASMS for lead is composed of three parts. The first part is a questionnaire and doctor’s review of lead-related symptoms, signs and exposure history. The second part is a clinical laboratory examination including a BLL examination. The third part consists of documentation of the worker’s personal information including name, Residence Registration Number (RRN: a unique 13-digit number assigned to all Korean citizens), gender, birth-date, first work-date at the lead-associated work position, company information on the type of industry as classified by the Korean Standard Industrial Classification code [KSIC] and the total number of workers. All of the above information has been electronically reported to KOSHA from medical institutes since 2000. Using this electronic data, we constructed the cohort for lead-exposed workers who had an ASMS including lead from January 1, 2000, to December 31, 2004. Vital status (death and cause of death) from 2000 to 2008 were identified by the KNSO, a registry estimated to achieve greater than 99% registration of deaths: cause of death was available beginning in 1992. KNSO records provide the RRN, cause of death (Korea Classification of Disease and Cause of Death, 5th edition, which is a system very similar to the International Classification of Disease and Cause of Death, 10th edition) [13] and date of death. Lead-exposed workers were matched to the KNSO database using the RRN.

### Exposure Assessment

Individual biological exposure assessments for BLL were conducted through the ASMS from January 1, 2000, to December 31, 2004. Each cohort member’s BLLs varied from one to five, depending on how many times the worker underwent the ASMS including lead during this period. We could not identify the cumulative exposure level for individual exposed workers, so we used median BLLs as surrogates of cumulative exposure. A subgroup of the expert members of the Association for Occupational and Environmental Clinics (AOEC) recommend that workers avoid further occupational lead exposure if two successive BLLs over a 4-week interval are ≥ 20 μg/dl and that the workers maintain BLLs < 10 μg/dl to avoid long term risks [14]. Schwartz and Hu favor limits that keep BLLs < 20 μg/dl to prevent the acute effect of the recent dose [15]. So, we classified each BLL into 3 categories: <10 μg/dl, 10 to 20 μg/dl and ≥20 μg/dl.

Other carcinogenic metal exposure (exposure or non-exposure) was classified by whether the worker had undergone the ASMS including cadmium, chromium, nickel and arsenic in the same period. Entry to the cohort was defined as the first medical check-up date. The cohort exit was defined as the date of death or December 31, 2008, whichever came first. Death causes were classified according to KCD-5: all cause of death, all non-malignant death (all codes except C00-C97), infection (A00-B99), Endocrine (E00-E90), circulatory (I00-I99), ischemic heart disease (I00-I25), cerebrovascular disease (I60-I69), respiratory disease (J00-J99), digestive disease (K00-K93), injury, poisoning and external causes (SOO-T98), intentional self-harm (X60-X84), total cancer (C00-C97), stomach cancer (C16), colon & rectum cancer (C18-20), liver & intrahepatic duct cancer (C22), gall bladder cancer (C23), cancer of other and unspecified parts of the biliary tract (C24), lung cancer (C34), breast cancer (C50) and leukemia (C91-93).

### Statistical analysis

The t-test and the chi-squared test were used to know the difference of general characteristics according to the subject’s gender (Table 1).

**Table 1:** General Characteristics of Lead-Exposed Workers.

Characteristics		Male		Female	
		N	%	N	%
**Total**		**54,788**	**67.6**	**26,279**	**32.4**
Age in 2000	**≤ 20**	**6,958**	**12.7**	**7544**	**28.7**
	**20–29**	**21709**	**39.6**	**6874**	**26.2**
	**30–39**	**14970**	**27.3**	**7122**	**27.1**
	**40–49**	**8880**	**16.2**	**4246**	**16.2**
	**50≤**	**2271**	**4.1**	**493**	**1.9**
	**Mean±S.D.** *	**31.1±9.8** *		**28.9±10.5** *	
Year of initial employment	**≤1979**	**2,379**	**4.3**	**23**	**0.1**
	**1980–89**	**9,466**	**17.3**	**654**	**2.5**
	**1990–99**	**22,054**	**40.3**	**11,710**	**44.6**
	**2000≤**	**20,889**	**38.1**	**13,892**	**52.9**
Age at initial employment	**<20**	**8,061**	**14.7**	**9,350**	**35.6**
	**20–29**	**34,532**	**63.0**	**5,157**	**19.6**
	**30–39**	**8,397**	**15.3**	**7,949**	**30.2**
	**40 ≤**	**3,798**	**6.9**	**3,823**	**14.5**
	**Mean±S.D.** *	**26.8±7.4** *		**28.2±9.8** *	
Duration of employment	**<10**	**27,798**	**50.7**	**18,950**	**72.1**
	**10–19**	**17,203**	**31.4**	**6,888**	**26.2**
	**20 ≤**	**9,787**	**17.9**	**441**	**1.7**
	**Mean±S.D.** *	**12.3±7.2** *		**8.7±3.6** *	
Survival status*	**Live**	**54,365**	**99.2**	**26,185**	**99.6**
	**Death**	**423**	**0.8**	**94**	**0.4**
Blood lead level(ug/dl)	**<10**	**41,153**	**75.1**	**23,037**	**87.7**
	**10–20**	**8,908**	**16.3**	**2,750**	**10.5**
	**≥ 20**	**4,727**	**8.6**	**492**	**1.8**
	**Mean±S.D.** *	**8.8±8.5** *		**5.8±5.4** *	
**Other metal** * **exposure**	**Exposure**	**12,747**	**23.3**	**568**	**2.3**
	**Non-exposure**	**42,041**	**76.7**	**25,711**	**97.8**

Other metal (chromium, arsenic, nickel, cadmium) exposure, *S*.*D*., standard deviation;

*p<0.05.

The Relative Risk [RR] and 95% Confidence Interval [CI] were calculated using Cox proportional hazard models. Each blood lead category’s (10–19 μg/dl and ≥20 μg/dl) relative risks were compared with the reference category (<10 μg/dl). We used the following variables as covariates: age in 2000 (20–29, 30–39, 40–49, 50≤) and other carcinogenic metal exposure (exposure or non-exposure) such as arsenic, cadmium, chromium and nickel. The analyses were calculated separately according to the subjects’ gender. These analyses were performed using the Windows-based SPSS statistical package (version 20.0; SPSS, Chicago, IL). More than three of each cause of death category were analyzed to ensure statistical power (Tables 2 and 3).

**Table 2:** Cause–specific mortalities of male workers by BLLs.

Cause of death	<10ug/dl		10–20 ug/dl		≥ 20 ug /dl	
	Death(n)	Relative risk	Death(n)	Relative risk(95%CI)) *	Death(n)	Relative risk(95%CI) *
All-causes	286	1.00	74	0.97(0.75–1.25)	**63**	**1.36(1.03–1.79)** ^†^
Non-malignant death	197	1.00	53	1.06(0.78–1.44)	46	0.95(0.56–1.51)
Infection	5	1.00	**5**	**3.73(1.06–13.06)** ^†^	1	1.22(0.14–10.72)
Endocrine disease	4	100	1	0.84(0.09–7.62)	**3**	**4.25(0.90–20.04)** ^‡^
Circulatory disease	29	1.00	8	0.98(0.45–2.16)	**10**	**1.99(0.95–4.15)** ^‡^
Ischemic Heart disease	12	1.00	4	1.12(0.36–3.52)	4	1.74(0.55–5.54)
Cerebrovascular disease	9	1.00	3	1.17(0.31–4.36)	3	1.90(0.50–7.28)
Respiratory disease	5	1.00	2	1.46(0.28–7.49)	0	-
Digestive disease	15	1.00	4	1.02(0.34–3.11)	**8**	**3.23(1.33–7.86)** ^†^
Injury, poisoning & external cause	116	1.00	23	0.83(0.53–1.31)	17	1.12(0.67–1.87)
Intentional self-harm	13	1.00	5	1.47(0.52–4.13)	**6**	**2.92(1.09–7.81)** ^†^
Total cancer	89	1.00	21	0.78(0.48–1.25)	17	0.95(0.56–1.61)
Stomach cancer	22	1.00	4	0.66(0.23–1.92)	3	0.80(0.23–2.71)
Colon & Rectum cancer	6	1.00	0	**-**	2	1.86(0.35–9.79)
Liver & intrahepatic duct cancer	21	1.00	3	0.48(0.14–1.61)	7	1.72(0.72–4.14)
Other and unspecified parts of biliary tract cancer	5	1.00	1	0.65(0.08–5.62)	2	1.92(0.36–10.31)
Bronchus and lung cancer	19	1.00	5	0.79(0.29–2.13)	2	0.46(0.10–2.01)
Leukemia	4	1.00	1	0.65(0.07–5.93)	0	**-**

Reference group is the lead level less than 10 μg/dl;

*The multivariate model includes the following covariates: age (*≤*20, 20–29, 30–39, 40–49, ≥50), other metal (chromium, arsenic, nickel, cadmium) exposure (exposure or non-exposure).

^†^p<0.0.5,

^‡^p<0.1.

**Table 3:** Cause–specific mortalities of female workers by BLLs.

	<10ug		10–20 ug/dl		≥ 20 /dl	
Cause of death	Death(n)	Relative risk	Death(n)	Relative risk (95% CI))*	Death(n)	Relative risk (95% CI)) *
All-causes	72	1.00	19	**1. 93(1.16–3.20)** ^†^	3	1.30(0.41–4.16)
Non-malignant death	53	1.00	**10**	**1.93(0.96–3.97)** ^‡^	1	0.99(0.13–7.19)
Circulatory disease	11	1.00	2	1.26(0.28–5.68)	0	-
Cerebrovascular disease	6	1.00	0	-	0	-
Respiratory disease	2	100	1	3.49(0.31–39.05)	0	-
Digestive disease	2	1.00	1	3.66(0.33–40.70)	0	**-**
Injury, poisoning & external cause	11	1.00	**4**	**2.87(0.91–9.01)** ^‡^	1	4.44(0.57–34.90)
Intentional self-harm	8	1.00	0	**-**	0	-
Total cancer	32	1.00	**9**	**1.89(0.95–4.20)** ^‡^	2	1.68(0.40–7.13)
Stomach cancer	4	1.00	1	1.82(0.20–16.36)	0	-
Colon & Rectum cancer	1	1.00	**2**	**13.42(1.21–149.4)** ^†^	0	-
Liver & intrahepatic duct cancer	8	1.00	1	0.83(0.10–6.56)	0	-
Gall bladder cancer	3	1,00	0	**-**	0	**-**
Bronchus and lung cancer	2	1.00	**3**	**10.45(1.74–62.93)** ^†^	**1**	**12.68(1.69–147.86)** ^†^
Breast cancer	6	1.00	0	**-**	0	**-**

Reference group is the lead level less than 10 μg/dl;

*The multivariate model includes the following covariates: age (*≤*20, 20–29, 30–39, 40–49, ≥50), other metal (chromium, arsenic, nickel, cadmium) exposure (exposure or non-exposure).

^†^p<0.0.5,

^‡^p<0.1.

## Results

### General characteristics of the cohort by gender

81,067 workers had the ASMS for BLL more than once from January 1, 2000, to December 31, 2004. The study population of 81,067 workers was followed for 552,166 person-years. The average age of male workers (**31.1±9.8**) in 2000 was statistically higher than that of female workers (**28.9±10.5**) and the average tenure of male workers (**12.3±7.2**) was statistically higher than that of female workers (**8.7±3.6**). The average age at initial employment of female workers (**28.2±9.8**) was higher than that of male workers (**26.8±7.4**). The proportion of male workers with BLL of 10–20 μg/dl (16.3%) and ≥20 μg/dl (8.6%) was higher than in female workers. And the average BLL in male workers (**8.8±8.5** μg/dl) was higher than that of female workers (5**.8±5.4** μg/dl). The proportion of death in male workers (0.8%) was higher than female workers (0.4%) and the proportion of exposure to carcinogenic metals such as chromium, arsenic, nickel and cadmium among male workers (23.3%) was higher than that among female workers (2.2%) (Table 1). Though we only had some lead-exposed workers’ smoking histories, the smoking rate of lead-exposed male workers was 55.5% and the smoking rate of female workers was 2.5% in 2000–2001. Especially for female workers, smoking rate with BLL ≥ 10μg/dl (1.9%) was lower than with BLL <10 μg/dl (2.9%) in our study (data not shown).

### Cause–specific mortalities of male workers by BLLs

The RRs of all-cause mortality (RR 1.36, 95% CI 1.03–1.79), digestive disease (RR 3.23, 95% CI 1.33–7.86), and intentional self-harm (RR 2.92, 95% CI 1.07–7.81) was statistically higher in male workers with BLL ≥20 μg/dl than with BLL ≤10μg/dl. The RRs of infection was statistically higher in male workers with BLL 10–20 μg/dl than with BLL ≤10μg/dl BLL (RR 3.73, 95% CI 1.06–13.06). The RRs of male workers with BLL≥20 μg/dl were moderated significant higher than 10 μg/dl (p<0.1) in endocrine disease (RR 4.25, 95%CI 0.90–20.04), circulatory disease (RR 1.99, 95%CI 0.95–4.15). The RRs of male workers with BLL≥20 μg/dl were elevated than 10 μg/dl in infection (RR 1.22, 95%CI 0.14–10.72), ischemic heart disease (RR 1.74, 95%CI 0.55–5.54), cerebrovascular disease (RR 1.90, 95%CI 0.50–7.28), colon and rectal cancer (RR 1.86, 95%CI 0.35–9.79), liver and intrahepatic duct cancer (RR 1.72, 95%CI 072–4.14), although not statistically significant (Table 2).

### Cause–specific mortalities of female workers by BLLs

The RRs of female workers with BLL of 10–20 μg/dl were statistically higher than of those with BLL of 10μg/dl in all-cause mortality (RR 1.93, 95% CI 1.16–3.20) and colon & rectum cancer (RR 13.42, 95% CI 1.21–149.4). The RRs of female workers with BLL of 10–20 μg/dl (RR 10.45, 95% CI 1.74–62.93) and 20 μg/dl BLL (RR 12.68, 95% CI 1.69–147.86) were statistically significant in bronchus and lung cancer. The RRs of female workers with BLL of 10–20 μg/dl were moderately statistically (p<0.1) higher than those with BLL of 10μg/dl in non-malignant death (RR 1.93, 95% CI 0.96–3.97), total cancer (RR 1.89, 95% CI, 0.95–4.20) injury poisoning, and external causes (RR 2.98, 95% CI 0.91–9.01). The RRs of female workers with 10–20 μg/dl BLL in circulatory disease (RR 1.26, 95% CI, 0.28–5.68), respiratory disease (RR 3.49, 95% CI 0.31–39.05), digestive disease (RR 3.66, 95% CI 0.33–40.70), total Cancer (RR 1.89, 95% CI 0.95–4.20), and stomach cancer (RR 1.82, 95% CI 0.20–16.36) were elevated but were not statistically significantly higher than 10 μg/dl. The RRs of female workers with 20 μg/dl BLL in all cause mortalities (RR 1.30, 95% CI 0.41–1.46), injury poisoning, external causes (RR 4.44, 95% CI 0.57–34.90), and total cancer (RR 1.68, 95% CI 0.40–7.13) were elevated, although not statistically significant (Table 3).

## Discussion

Steenland and Boffetta proposed that the most likely indication associating lead with cancer is lung cancer [16]. Indeed, Vainio said that long-term, high exposure to lead compounds is associated with an increased risk of lung cancer [17], and Anttila et al. observed a 1.8-fold increase in lung cancer incidence for study participants who had a BLL of ≥1.0 μmol/l [18]. In our study, increased lung cancer mortality was only observed in female workers. We think that this gender difference has many causes. First, the significant higher risk of lung cancer in women (10–20 μg/dl and 20 μg/dl) might not be a smoking effect. Because, although we did not control the individual smoking history in the current study, the smoking rate of females with BLL ≥ 10μg/dl (1.9%) was lower than of those with <10μg/dl BLL (2.9%). Also, the smoking rate itself was very low in women. However, though the smoking rate of males with BLL ≥ 10μg/dl was lower than of those with <10μg/dl BLL, the men’s smoking rate itself was high (55.5%). So, in this study, the smoking effect on lung cancer mortality was not excluded in men. That is, higher lung cancer mortality in women with high BLLs might be affected by lead exposure not by smoking. Second, our study controlled for other carcinogenic metal exposure, so increased lung cancer mortality in female workers should be considered as a meaningful result. In addition, the authors speculate that the males were exposed to wide range of occupational sources than females like the Shanghai Men’s and Women’s Health Study Cohorts [19], so the association with lung cancer and BLL was not observed in our study. Control of individual smoking history and other confounders and a long-term follow-up study are needed in the future.

Wingren and Axelson conducted a case-control study of the risk of cancer for art-glass workers in Sweden, and significantly increased odds ratios were found for total cancer, stomach cancer, lung cancer and colon cancer [20]. For colon cancer, a clearly increasing trend in risk was seen with increasing use of antimony, and to some extent with increasing use of lead, the two elements were strongly correlated [20]. An increase of colon and rectal cancer was observed in female workers with 10–20 μg/dl BLL in our study. Lead exposure and the occurrence of colorectal cancer are not well studied and there are few related studies until now. So we can’t explain about increased risk of colorectal cancer related to lead exposure. Also, because we did not control the confounders such as diet, menopause, physical activity and other risk factors of colorectal cancer, we can’t exclude the effect of such risk factors. However in our study, about 50% of study subjects checked the body mass index (BMI) and total blood cholesterol, which are the major risk factors of colorectal cancer. The average BMI and Total cholesterol in female workers with BLL <10 μg/dl were higher than those of workers with BLL ≥10μg/dl in 2000–2005 (data not shown). This suggests that the increased colorectal cancer mortality in women in our study might not be related to the cholesterol but rather to lead exposure. Our result should be followed further and confounders including physical activity and other risk factors need to be controlled.

Among whites in the National Health and Nutrition Examination Survey II (NHANES II), a dose-response relationship between BLL and total cancer was found at highest BLL in females but not in males [21]. Similar to this previous study, our study showed a moderate significant increase in total cancer in female workers with BLL of 10–20 μg/dl (RR 2.03, 90% CI 1.09–3.79), while the effect was not observed in male workers.

Chowdhury et al. found a statistically significant positive trend in heart disease mortality with increased blood lead category in internal comparisons among men in a lead surveillance program [22]. The findings of NHANES II and NHANES III showed a statistically significant increase in cardiovascular disease with increasing BLL [5,23,24]. The British Regional Heart study showed that mean BLLs are somewhat higher in individuals who subsequently have a heart attack or stroke [25]. However, after allowance for cigarette smoking and town of residence, this association was no longer statistically significant [25]. No relationship was found between BLL and 8-year incidence of coronary heart disease after both univariate and multivariate analysis among elderly men in Netherlands [26]. The systemic review of lead exposure and cardiovascular disease indicates that the evidence is suggestive but not sufficient to infer a causal relationship of lead exposure with clinical outcomes (cardiovascular, coronary heart disease, stroke mortality and peripheral disease) [23]. Vaziri suggested that lead exposure can promote hypertension, arteriosclerosis, atherosclerosis, thrombosis and cardiovascular disease [27,28]. Our study showed a moderate significant association (RR 2.01, 90% CI, 1.08–3.73) with circulatory disease mortality of male workers. Analyzing systolic and diastolic blood pressure (SBP and DBP) of around 50% of our study subjects, SBP and DBP were significantly increased by BLL. However, BMI and total cholesterol were significantly lower with BLLs of 10–20 μg/dl than with BLLs of < 10 μg/dl. So in this study, increased cardiovascular disease mortality according to BLL suggests an association with occupational lead exposure.

We found a moderate significant association (RR 1.29, 90% CI 1.17–15.7) with endocrine disease mortality of male workers. And the main cause of endocrine disease mortality was diabetes mellitus (77.8%) in our study. Some heavy metals such as zinc, arsenic, cadmium, mercury and nickel may play an important role in diabetes mellitus as environmental risk factors [29]. Lead-exposed worker showed more obesity, higher fasting glucose, total cholesterol, lactate dehydrogenase, and uric acid levels and lower levels of low density lipoprotein cholesterol than non-exposed workers in United Arab Emirates. [30] It is well known that high levels of exposure to occupational or environmental toxicants, such as lead, mercury, and cadmium, can cause specific nephropathies [31]. In a recent study, Huang et al. indicated that body lead burden and BLL, even at low level, are important risk factors for progressive diabetic nephropathy and these associations were strong, dose-dependent, and consistent, even after comprehensive adjustment for other covariates [32]. Whether lead can cause diabetes mellitus is unknown, but lead may be an important risk factor of progressive diabetic nephropathy, so the increased endocrine mortality in our study is notable.

Adults with a low socioeconomic status had a higher burden of lead exposure. Specifically, after adjusting for age, race, ethnicity, and gender, the odds ratio of having a blood lead level of 5 μg/dL or more was 1.74 (95% CI, 1.23–2.46) for adults with an annual household income of less than $20,000, 2.62 (95% CI, 1.95–3.52) for adults lacking health insurance and 2.67 (95% CI, 1.83–3.90) for adults with less than a high school education [29]. Suicide risk was strongly associated with mental illness, unemployment, low income, marital status, and family history of suicide and the effect of most risk factors differed significantly by gender in Denmark [33]. In the National Longitudinal Study of Adolescent Heath, suicide attempt was linearly associated with income (p<0.1) in American adolescents [34]. The geometric means of BLL were higher in the “Manufacture of Electrical Equipment” including lead storage batteries and in the “Manufacture of Rubber and Plastic Products” including the process of manually mixing stabilizers while manufacturing Poly Vinyl Chloride products than in other divisions in Korea [35]. High lead exposure is more likely to occur in small plants where the workplace tends to be more poorly controlled compared to large plants [36]. The authors speculate that male workers who worked at high lead-exposure worksites were likely to have lower socioeconomic status and higher chances of unemployment than for low BLL workers. Low income, unemployment and low socioeconomic status of male lead-exposed workers were associated with intentional self-harm. Also, previous studies showed that BLL was associated with major depression and other psychological disorders. [37,38, 39]. Among previous studies, one study [37] included females, others did not. Although one study has not been published yet (currently under review), analyzing the same data as our study, significant increment trend of hazard ratios for overall mental disorders, mental disorder and behavior disorder due to psychoactive substance use (F10-F19), mood (affective) disorders (F30-F39) and unspecified mental disorder (F99) according to BLLs was observed in male lead exposed workers. Increased suicide mortality of males with over 20 μg/dl BLL was likely related to this increment. Further study on BLLs associated depression in females is needed.

The RR of male workers with BLL ≥20 μg/dl in digestive disease was statistically higher than with BLL ≤10μg/dl in our study. The most common causes of death with BLL ≥20 μg/dl were liver cirrhosis and hepatic failure (87.5%) in this study. Multiple etiologic factors contribute to the development of liver cirrhosis [40], Hepatitis B antigen positivity was not affected by social economic status but by male gender [41] and hepatitis C is related with old age but not gender in South Korea [42]. Drinkers of lower economic status have a higher prevalence of alcohol- related problems [43, 44]. Recently, Mazumdar and Goswami showed that chronic lead exposed workers had higher liver enzyme levels than controls [45]. They said that chronic exposure to lead increases oxidative stress, and may lead to necrosis of liver cells, and hepatocellular injury [45]. The authors suggest that lead exposure may affect the progress of liver cirrhosis, as the old and male workers who had high BLL were more likely to have alcohol- and hepatitis- related problems than those who had low BLL.

In this study, there were slight (not statistically significant) elevations of male workers in infection (RR 1.22, 95%CI 0.14–10.72), cerebrovascular disease (RR 1.90, 95%CI 0.50–7.28), colon and rectal cancer (RR 1.86, 95%CI 0.35–9.79), and liver and intrahepatic duct cancer (RR 1.72, 95%CI 072–4.14). And there were slight elevations of female workers with 10–20 μg/dl BLL in circulatory disease (RR 1.26, 95% CI 0.28–5.68), respiratory disease (3.49, 95% CI 0.31–39.05), digestive disease (RR 3.66, 95% CI, 0.33–40.70), and stomach cancer (RR 1.82, 95% CI 0.20–16.36) compared with <10 μg/dl BLL. In addition, there were slight elevations of female workers with ≥20 μg/dl BLL in all cause mortalities (RR 1.30, 95% CI 0.41–1.46), injury poisoning, external causes (RR 4.44, 95% CI 0.57–34.90), and total cancer (RR 1.68, 95% CI 0.40–7.13). Because, there were small numbers of deaths in males and females, further follow-up of our study is needed.

Our study has several strengths. The first strength is the inclusion of all cohort members’ median BLLs measured one to five times. Secondly, the authors inspected exposure to other carcinogenic metals which could be confounding factors and could partly explain other confounding factors such as smoking, alcohol, BMI and total cholesterol (data not shown). Finally, this cohort is representative of Korean lead-exposed workers because the sample size was large.

Despite these strengths, our study has several limitations. First, using only electronic data collected by ASMS (secondary data), we could not investigate potential confounders such as dietary habits, physical activity, education, socioeconomic status, etc. The second limitation of our study was the lack of complete information on exposure history, including previous lead exposure prior to cohort construction. Lead-exposed workers who became severely ill or chronically disabled may not be included in this cohort as they may have left their employment prior to the cohort construction (the healthy worker survivor effect), which also might have affected the study results. Third, the BLL in our study was based on the median of annual BLLs, so it may not accurately reflect cumulative exposure to lead but may only provide an approximation it. Fourth, there was a significant increase of colorectal cancer in female workers, but in the current study, we observed just three cancer deaths among females. Only two female cases were observed in 10–20 μg /dl BLL. Hence, although there was a significant increasing risk of colorectal cancer in 10–20 μg/dl BLL, careful attention to such limitations were needed when interpreting our current results. Finally, our study followed the ICD-10 codes and each subcategory is composed of the diseases in the same anatomical site, which is likely to reduce the competing risk due to sub-cause analyses. Because we used secondary data in this study, we could not control competing risk factors. This is a major limitation of our study.

The important finding of this cohort is that higher BLL is statistically associated with lung cancer mortality in female workers. The increased suicide of males with ≥20 μg/dl BLLs, which might be caused by major depression, might be associated with higher lead exposure. In this study, the kinds of BLL-associated mortality differed by gender. This might be caused by gender differences in occupational exposure and distribution of confounders. Further studies are needed to elucidate this gender difference after controlling occupational and other confounders. Also, long-term follow-up of this cohort is needed to reveal causal relationships between specific causes of death and lead exposures. So, ASMS for lead exposure should be sustained to follow up morbidity & mortality and to create policies that reduce lead exposure in the workplace.

## Abbreviations

BLL: blood lead level
RR: relative risk
CI: confidence interval
